# Timber isoscapes. A case study in a mountain area in the Italian Alps

**DOI:** 10.1371/journal.pone.0192970

**Published:** 2018-02-16

**Authors:** Yuri Gori, Ana Stradiotti, Federica Camin

**Affiliations:** 1 Department of Food Quality and Nutrition, Research and Innovation Centre, Fondazione Edmund Mach (FEM), San Michele all’ Adige, Trento, Italy; 2 Stable Isotopes Center, Institute of Biosciences, São Paulo State University (UNESP), Botucatu Campus, Brazil; Austrian Federal Research Centre for Forests BFW, AUSTRIA

## Abstract

**Background:**

Local timber is still one of the main sources of work and income for mountain communities. However, illegal logging is a major cause of deforestation in many countries and has significant impacts on local communities and biodiversity. Techniques for tracing timber would provide a useful tool to protect local timber industries and contribute to the fight against illegal logging. Although considerable progress has been made in food traceability, timber provenance is still a somewhat neglected research area. Stable isotope ratios in plants are known to reflect geographical variations. This study reports accurate spatial distribution of δ^18^O and δ^2^H in timber from north-eastern Italy (Trentino) in order to trace geographical origin.

**Methodology and principal findings:**

We tested the accuracy of four kriging methods using an annual resolution of δ^18^O and δ^2^H measured in *Picea abies*. Pearson’s correlation coefficients revealed altitude to be the most appropriate covariate for the cokriging model, which has ultimately proved to be the best method due to its low estimation error.

**Conclusions:**

We present regional maps of interpolated δ^18^O and δ^2^H in *Picea abies* wood together with the 95% confidence intervals. The strong spatial structure of the data demonstrates the potential of multivariate spatial interpolation, even in a highly heterogeneous area such as the Alps. We believe that this geospatial approach can be successfully applied on a wider scale in order to combat illegal logging.

## Introduction

Exploitation of local timber, especially in Alpine regions, is of central importance to the survival of mountain communities and local economies. As markets have expanded from local to global, many timber species previously in demand on the local market have now been superseded by cheaper, imported timber species. We hold that the use of local timber should be encouraged in order to reduce the environmental impact of transportation. Furthermore, given the global market, consumers are increasingly interested in knowing the geographical origin of timber. Recognising the added value of local timber with its short supply chain and benefits to the local economy, they are often prepared to meet the higher costs of these products for ecological, social-ethical and ideological reasons. This is particularly significant for an Alpine region like Trentino, with 60% of its surface area covered with forest.

Deforestation continues at an alarming rate in many countries and each year an area of about 130000 km^2^ (the size of Greece) is lost, which not only has adverse effects on local economies but also threatens irreplaceable biodiversity, increasing the rate of species extinction, and contributes to global warming [1].

Deforestation is obviously linked to illegal logging. Moreover, illegal logging is also associated with other negative externalities, such as the introduction and spread of harmful pests, violation of ownership, tax evasion and corruption [2].

Illegal logging and illegal trade take place in tropical forests as well as in non-tropical regions, such as Georgia and Albania [3,4], where it accounts for between 85% and 95% of overall timber production. Global economic losses from the illegal timber trade are estimated at around US$10 billion per annum [5,3]. However, in the recent past the illegal timber trade has received much attention from NGOs and policymakers through various legislative measures (e.g., EUTR, the US Lacey Act).

The development of a suitable method able to identify geographical provenance would be a very useful tool in the fight against illegal logging and would consequently protect local economies [2]. For example, it could be used to verify whether a timber product has been harvested from a legitimate source (e.g., because it comes from an area where forest certification is mandatory). There are currently two methods that are mainly used as proof of tree provenance: i) DNA fingerprinting, by comparing chloroplast DNA of wood of unknown origin with chloroplast DNA of a reference region [6–9], and ii) Dendroprovenancing [10].

Tree ring-width series have often been used to estimate the likely origin of unknown wood. Because trees growing close to each other show similar growing patterns, comparison can be made of the t-values of the tree-ring chronologies of woods of known and unknown origins [10]. However, climate and ecological variability on a local scale, silvicultural treatment, fungal disease [11,12] and insect attack [13] have a strong influence on tree-ring width chronologies, affecting subsequent proof of traceability. For a comprehensive review of dendroprovenancing, see Bridge M. [14].

Several protocols to extract DNA from wood have so far been developed. Given that DNA fingerprints reflect patterns of genetic diversity within and between populations, [10, 15,16] it can be successfully used in the case of natural regeneration and when species show a clear spatial genetic structure in their native area, but may, on the other hand, fail in the case of artificial regeneration from seeds of unknown origin. Finally, Sheikha et al. [17] recently proposed a successful molecular technique (PCR-DGGE) to characterise fungi flora as a tool for tracing the geographical origin of timber. This method is simple, rapid and cheap, but may fails to describe spatial structure.

On the other hand, stable isotope ratios reflect geographical variation because they are highly dependent on the isotopic composition of meteoric precipitation, which in turn is related to geographical location [18]. Of the various available techniques [19], stable isotope ratio analysis has proved to be a powerful method for identifying the provenance of agricultural products, such as cheese [20], wine, orange juice [21], meat [22,23], olive oil [24], milk [25] and even drugs [26].

At present, stable isotope ratios have only been applied to examine differences between timbers on a macro or mega scale (over 5,000 km). For example, using δ^13^C and δ^18^O values of bulk wood, Horacek et al. [27] were able to discriminate between Siberian larch and European larch on a continental scale, while Keppler et al. [28] found a strong correlation between the δ^2^H values of the lignin methoxyl groups and the OIPC (Online Isotope Precipitation Calculator; accessible at http://waterisotopes.org/) δ_2_H values of meteoric water on a global scale. In our pilot study [29], we showed that δ^18^O and δ^2^H values in *Picea abies* bulk needles are a safe proxy for identifying geographical origin, as they vary according to geographical position, even on a regional scale.

Spatial variability in the isotopic composition of organic matter is strongly patterned due to the underlying processes being spatially autocorrelated, producing spatially coherent *isoscapes*. The word *isoscapes* was coined by West and colleagues and is a merging of the terms “isotope” and “landscapes”, thus defining the geospatial predictive power of stable isotopes [30]. It is noteworthy that there has been a large increase in studies focusing on isoscapes in the last decade [31], mostly in the fields of biogeochemistry, climate dynamic models, water cycles, animal migration and food traceability [24, 32–34]

Spatial interpolation provides a powerful tool for estimating the value of a variable where data are not available by generating a regular grid surface that takes into account the spatial variability of the data. Among the various interpolation methods, kriging is a generic term for several techniques that provide best linear unbiased predictions (BLUP), best in the sense of minimum variance [35]. Kriging was developed and formalized in the early ‘60s by Krige and Matherons, and since then it has been widely applied in various fields, such as geology, climatology, soil science, animal ecology and remote sensing [36]. Associated error maps with confidence intervals can also be constructed using kriging, which is not possible with other tools, such as the inverse distance weighted method.

Although geospatial modelling has recently been successfully used in food authentication [20,21,24,31], to our knowledge, no isoscape studies have been carried out with the aim of estimating timber provenance.

From a geostatistical point of view, the general assumption in geospatial modelling is that the data are spatially continuous, an assumption that is met since Bowen [37,38] published spatial continuity isotope maps for δ^2^H and δ^18^O. Moreover, when there are sufficient data to compute a variogram, kriging also provides good interpolation for sparse data [36].

In this work, we have used kriging procedures to study the spatial distribution of δ^2^H and δ^18^O in Norway spruce wood (*Picea abies*) using a set of 151 sampling areas in a south-eastern Alpine region (Trentino, Italy). We compared the robustness of δ^2^H and δ^18^O surfaces produced in 4 kriging modes: a) ordinary kriging, b) universal kriging with linear trend, c) universal kriging with quadratic trend, d) cokriging. Leave-one-out cross-validation was finally applied to the models in order to find the best one for prediction.

Our main goals were:

1. to analyse and quantify the uncertainty characterizing the spatial distribution of δ^2^H and δ^18^O in Norway spruce wood, and thus assess the suitability of a spatial model;
2. to develop δ^2^H and δ^18^O isotope maps of Norway spruce in Trentino with the aim of enhancing the value of local timber and preventing illegal logging.

We believe that in the future will be possible to apply these isoscapes effectively on a continental or global scale to identify the likely area of origin of wood, thereby contributing to combatting illegal logging.

## Materials and methods

### Ethics statement

Our field studies did not involve endangered or protected species. The coordinates of the study locations are provided in S1 Table of the supporting informations.

We sampled four increment cores of 755 *Picea abies* in Trentino and no animals were involved in this study. Sampling was only conducted in alpine public forests where no permits for were required.

### Field sampling and cross-dating

**Fig 1:**
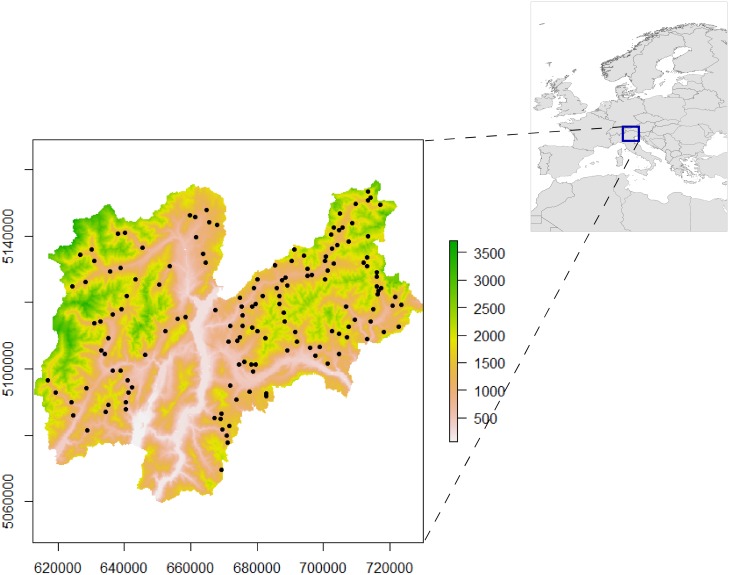
Location of the 151 sampling sites in Trentino Province. Altitude is represented by the white-green colour scale.

Field sampling was carried out in Norway spruce stands. In accordance with forest management planning in Trentino, we selected stands containing 20% or more Norway spruce trees. Of these stands, we selected 151 random locations (Fig 1) with elevations ranging from 547 to 1,974 m asl (GPS GeoXT 3000 series, Trimble, Sunnyvale, USA). At each site, five trees in a plot of about 2,000 m^2^ were selected for sampling. From September to December 2015, four increment cores were extracted at 90° from each other at breast height using a 0.5 cm diameter Pressler increment borer. To prevent isotopic contamination, no lubricants, markers or sandpaper were used during field sampling and in taking the subsequent dendrochronological measurements.

The cores of all trees were prepared with a core-microtome [39] to render the features of the rings clearly visible under magnification, and standard dendrochronological methods were used to cross-date [40]. Briefly, ring-width measurements were made to the nearest 0.01 mm on each core using LINTAB measuring equipment (Frank Rinn, Heidelberg, Germany). Tree-ring widths of each curve were plotted and cross-dated visually, and then statistically by (a) Gleichläufigkeit, i.e., the percentage agreement in the signs of first-differences between two time series, and (b) a t-test, which determines the degree of correlation between the curves. Cross-dating quality was checked using the COFECHA programme [41] as a standard protocol for quality control.

After dating, the cores were prepared for isotopic analysis. Annual wood rings formed in 2013 and 2014 were split with a razor under a stereoscope (Leica MS5). Finally, tree rings from the same site (five trees per site) were pooled. The samples were then ground in liquid nitrogen in accordance with Laumer et al.’s [42] method, slightly modified by Gori et al. [13].

### Predictor covariate

According to previous studies [43–45], when a covariate is easier and cheaper to measure and more densely sampled, cokriging may improve the accuracy of the interpolation. In order to reduce the risks associated with multiple comparisons [46], we selected only the proxies of known importance for stable isotope fractionation in trees [47]. For this reason, a total of 4 covariates (summer temperature, altitude, solar radiation and canopy cover, as reported in the following sections) were chosen according to:

- a clear feature-space correlation with the δ^18^O and δ^2^H of wood
- availability of more homogeneous and dense data

#### Summer temperature

As we reported in a previous study [29], isotope fractionation of *P*. *abies* in the Alps is closely related to summer temperature. This proxy was obtained with MODIS LST, following the method described by Neteler [48]. Briefly, temperature data were reconstructed using daily Land Surface Temperature (LST) maps (available from https://wist.echo.nasa.gov). Datasets were compiled using the MOD11A1 Daily LST sensor (carried by the Terra satellite), which provides very accurate daily minimum, maximum and mean temperatures in a grid format. Clouds and other atmospheric disturbances were filtered using an algorithm developed by Neteler [48] for GRASS-GIS (GRASS-GIS, version 7.0, available at http://grass.osgeo.org, GRASS Development Team, 2014).

#### Spatial and structural variables

In addition to environmental factors, altitude, solar radiation and canopy cover may have a strong influence on plant isotopic composition [29,49–52]. These variables also have a strong spatial structure. Moreover, in Trentino these proxies may be derived from LiDAR data (Laser Imaging Detection and Ranging, acquired in October, November and December 2006 using an Optech ALTM3100C laser system; see S1 Dataset for detailed parameters), which are available at a high spatial resolution and under the Creative Commons Attribution (http://www.territorio.provincia.tn.it/portal/server.pt/community/lidar/847/licenza_d%27uso/255622).

Solar irradiance, expressed in W/m_2_ for the entire year, was computed using the LiDAR-derived Digital Elevation Model (DEM, a regular grid surface with a spatial resolution of 1 m^2^, available at http://www.territorio.provincia.tn.it/portal/server.pt/community/lidar/847/lidar/23954) using the *r*.*sun*.*daily* modules implemented in GRASS-GIS. Altitude was extracted from the same DEM.

Since radiation for photosynthetic CO_2_ assimilation is strongly affected by canopy cover, we also computed this variable as a further indicator of available energy for photosynthesis [53]. Canopy cover, expressed as a percentage, was estimated using the LiDAR-derived canopy height model (LiDAR-CHM, available on the above-mentioned website) following the method described by Torresan et al. [54]. Briefly, in a first step we reclassified the CHM: assuming that vegetation under 2 m was formed only by shrubs, values less than 2 m were set to 0, while values above 2 m were set to 1, thereby obtaining a binary raster. For each cell of the binary raster, we calculated the sum of the pixel values within a neighbourhood radius of 20 m to produce a new raster (using the *r*.*neighbors* module implemented in GRASS-GIS). Canopy cover was then computed as the ratio between the area of this new raster and 1,256.4 (i.e. the area of a circular plot of 20 m radius).

### Isotope analysis

Samples of about 0.30±0.5 mg of the dried ground wood were placed in silver capsules for δ^18^O and δ^2^H analysis. All samples were then oven dried (80°C) to remove water vapour, then stored in a desiccator until analysis. The analysis was performed according to the procedure described in [55]. Values are expressed according to the IUPAC protocol [56]. δ^2^H values were calculated against USGS lignin 54 (δ^2^H = -154.4 ‰, δ^18^O: +17.8‰) and 56 (δ^2^H = -44‰, δ^18^O: +27.23‰) through the creation of a linear equation [52]. A sample of NBS-22, a fuel oil that doesn’t exchange, and a control wood sample were routinely included in each analytical run as a check of system performance. In both cases, we obtained highly repeatable results over the 2 month running period (standard deviation < 2 ‰).

Measurement uncertainty, expressed as 1 standard deviation when measuring the control sample 10 times, was 0.4 ‰ for δ^18^O and 2 ‰ for δ^2^H.

### Statistical analysis

Descriptive statistics were computed for all the stable isotope series and explanatory variables. Prior to analysis, data were examined graphically and tested for normality (Shapiro-Wilk test), skewness and Kurtosis, since normal distribution of values is required for kriging [57].

The relationship between the stable isotope values and the explanatory variables was explored by Pearson’s correlation coefficient. Finally, the presence of spatial autocorrelation in the isotope values was tested by means of Moran’s *I*.

All statistical analyses were carried out with the R 3.2.2 programme [58] (see S1 Dataset).

#### Variogram and geostatistical analysis

We included 4 target spatial variables, i.e., δ^18^O and δ^2^H of the 2013 wood (δ^18^O_2013_, δ^2^H_2013_) and δ^18^O and δ^2^H of the 2014 wood (δ^18^O_2014_, δ^2^H_2014_).

Spatial variability was assessed by modelling experimental variograms. These efficiently synthesises spatial variability and are assumed to be useful for understanding the complexity of a spatial variable [59].

In order to obtain the best fitting model, we used the approach proposed by Webster and Oliver [60]. Briefly, we plotted the experimental variogram for the four spatial isotope variables with the average data spacing as lag distance, then for each variogram we used the 3–4 function, which seemed to have the best fit by eye, choosing from the most popular functions (i.e., spherical mode, exponential model, linear model and Matern model). Finally, we chose the one with the smallest residual sum of square (RSS) and lowest Akaike Information Criterion (AIC). *Sill* and *range* in each chosen variogram model were, respectively, the variance of the variables (δ^18^O and δ^2^H) and the distance at which the variogram reached the sill. The *nugget*, representing independent errors or variation on a small scale, was calculated as the mean variation at the mean sampling distance. The directionality of the spatial correlation (anisotropy), spatial heterogeneities and the presence of trends were investigated with the experimental directional variograms and variogram maps [35]. Linear and quadratic models [61] were adopted to remove the trend effect from the variogram (when present) and stabilize the variance.

Finally, we chose the covariate with the lowest AIC out of the above-mentioned explanatory variables in order to perform cokriging. Since the mean and the variance between the auxiliary variables and δ^18^O and δ^2^H were not the same, the covariate was standardized. In the case of cokriging, variograms were computed for both the variable and covariate and fitted using the same above-mentioned approach for ordinary kriging.

#### Cross-validation

We tested the robustness of four kriging methods, so that a total of 4 krige-interpolations were performed for each stable isotope series. The models were (1) ordinary kriging (*KriO*), (2) universal kriging with linear trend (*KriL*), (3) universal kriging with quadratic trend (*KriQ*), and (4) cokriging (*CoK*).

Leave-one-out cross-validation was finally performed in order to assess the performance of these spatial interpolations and choose the kriging model that best predicted δ^18^O and δ^2^H. Two statistics were then computed: the root mean squared error (RMSE) and the mean squared deviation error (MSDR), which is the mean of the squared errors divided by the corresponding kriging variances from the cross validation. RMSE is a good criterion for determining the most precise model among a series of models tested, but the RMSE should be the minimal as kriging minimizes the variances. If the variogram model is accurate, the mean expected squared error should be equal to the kriging variance. MSDR is regarded as the best diagnostic test for kriging [59,62]. Since the MSDR should ideally equal 1, we chose the final model for which the MSDR was closest to 1.

## Results and discussion

### Explanatory variables

The coordinates of the study locations together with the mean δ^2^H and δ^18^O values and the candidate covariates are provided in S1 Table. Since we pooled annual wood rings formed in 2013 and 2014 from the same sites (five trees per site), it is not possible to show local isotopic shifts at each location. Nevertheless, several studies confirm that a pool of 5 rings sampled at the same site is sufficient to obtain a representative site isotope signal [63]. Moreover, in the case of kriging, we would like to point out that local variability is not lost by pooling rings, but is instead preserved in the nugget value (see the “Variogram and geostatistical analysis” section above). Indeed, isoscapes are constructed by modelling the variogram curve, which relates the distance between 2 points to the difference in shift at that distance. For this reason, all the experimental points with approximately the same distance between them would contribute equally to defining the optimal model. In this sense, points with the same distance between them behave like ‘pseudo replicates’ and the model is robust against the presence of possible local outliers, which is feasible, especially in our study, since we undertook a very intensive field sampling (151 sampling areas).

Descriptive statistics were computed for all the data. Normality tests (reported in S2 Table) show the data to be normally distributed and only slightly skewed, so data transformation was not necessary.

**Fig 2:**
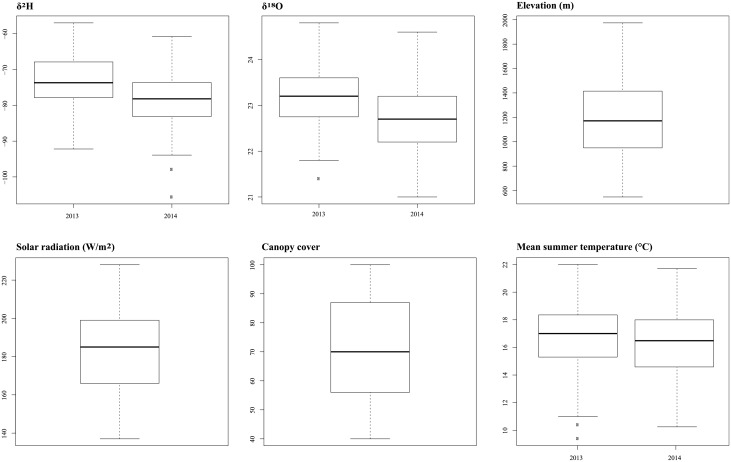
Box plots of δ^18^O and δ^2^H and the 4 candidate covariates.

The distribution was explored by box plot and the results are summarised in Fig 2. The δ^2^H and δ^18^O values in 2013 (δ^2^H_2013_, δ^18^O_2013_) were always higher than in 2014. Overall, the δ^2^H values ranged from -57.0‰ to -105.6‰, and the δ^18^O from 21.0‰ to 24.8‰. These finding are consistent with our previous research where we reported similar values for both δ^2^H and δ^18^O in three Norway spruce stands in Trentino [55], although we used different reference materials to normalise the data.

The relationship between δ^2^H and δ^18^O in wood and the candidate covariates was explored by Pearson’s correlation coefficients (Table 1).

**Table 1:** Coefficients of correlation between isotope ratios and explanatory variables.

	Altitude (m)	Mean summer temperature	Summer radiation	Longitude	Latitude
**δ**^**18**^**O**_**2013**_	-0.35***	0.21*		-0.25*	
**δ**^**18**^**O**_**2014**_	-0.65***	0.28**	-0.25*	-0.26**	-0.22*
**δ**^**2**^**H**_**2013**_	-0.39***	0.35***		-0.22*	-0.52***
**δ**^**2**^**H**_**2014**_	-0.3***	0.37***			-0.54***

* *p* < 0.05;

**p < 0.01;

*** *p* < 0.001

Values not significant at *p* < 0.05 are not reported.

Canopy cover was not reported since no correlations were found

The strongest correlation was obtained for altitude as a predictor of both δ^2^H and δ^18^O values (p < 0.001). Highly significant relationships between the δ^2^H and δ^18^O series and mean summer temperature were also found in some cases. Contrary to what we expected from our previous studies [29], we found only a weekly relationship between irradiance and δ^18^O in 2014 and no correlation with canopy cover. We do not have a clear explanation, but this discrepancy may be due either to different geographical scales (the previous study was carried out within a range of 600 km and the current one within a range of 120 km) or different plant materials (needles in the previous study, wood in the current one). On the other hand, solar radiation may vary considerably over a small scale because of “light gaps”, i.e., breaks in the forest canopy, for example, when a tree falls, and because canopy cover is affected by high microscale variation (i.e., high *nugget*) [53]. Solar radiation could, therefore, fail as a covariate on a local geographical scale. Finally, it is worth noting that some of the predictor variables may co-vary together. For example, mean summer temperature decreases along an altitude gradient because atmospheric pressure falls with increasing elevation, and for this reason altitude should be considered an independent variable (S4 Table show the relationships between all the variables via a correlation matrix).

The δ^18^O series correlated negatively with longitude (*p* < 0.005) and with latitude only in 2014. A more significant negative relationship was found only between δ^2^H and latitude. Depletion in ^2^H and ^18^O in organic matter is expected with increasing latitude, because ^2^H and ^18^O become gradually depleted as precipitating air masses travel from tropical regions toward the poles, a phenomenon known as the “latitude effect” and which is well reported in the literature [64].

As with our pilot study [29], we found a “longitude effect”, especially for the δ^18^O series. It should be born in mind that the western valleys of Trentino are often drier than the eastern valleys, so that the presence of a precipitation gradient from west to east satisfactorily explains the depletion in both δ^18^O and δ^2^H from east to west. The longitude effect has also been observed in the ^2^H of bulk Italian olive oils from the Tyrrhenian to the Adriatic coast [65].

The lowest AIC values were reported for altitude (detailed AIC values are shown in S3 Table for all the covariates), so this was used as covariate for the cokriging model.

Moran’s Index was computed on all the isotope ratios in order to investigate their spatial patterning: we found positive spatial autocorrelations for both δ^2^H and δ^18^O (p < 0.001).

Overall, these results are consistent with a strong influence of location on δ^2^H and δ^18^O, which other researchers have also reported [33]. This clear spatial dependence likely reflects the spatial structure of the meteoric water isotopic compositions [49].

### Variogram analysis and kriging models

**Fig 3:**
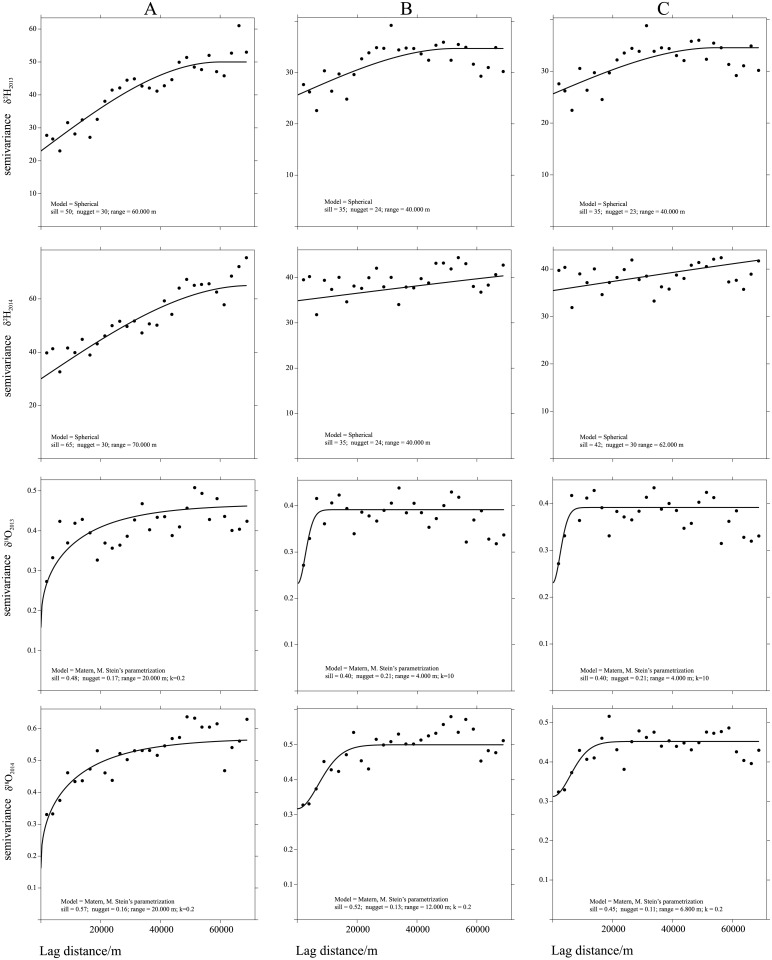
Variograms of δ^2^H and δ^18^O for 2013 and 2014. The A column shows those computed without trends, B indicates variograms detrended by a linear model, and C shows those detrended by a quadratic model. The lags have been binned over all directions and incremented in steps of 2,500 m. Solid lines show the models fitted to the variograms with the parameters reported in each panel. The fitted models were chosen on the basis of the smallest RSS, as reported in the text.

**Fig 4:**
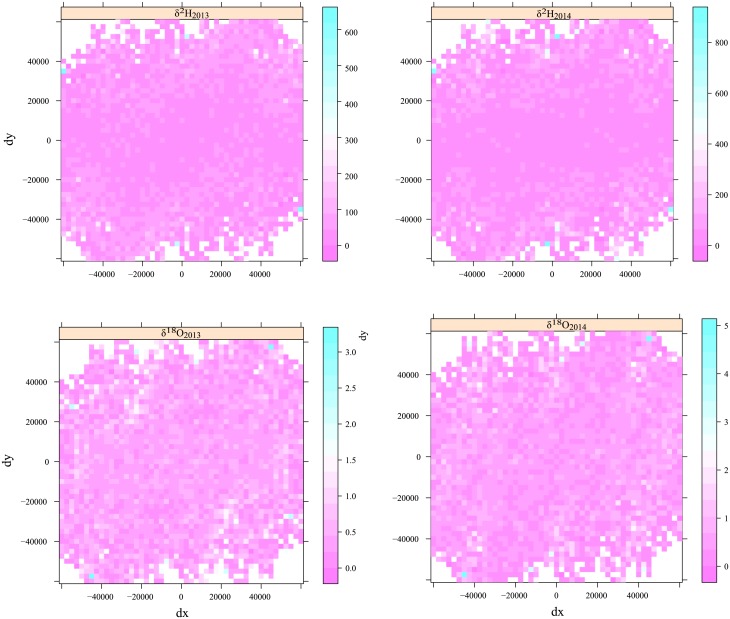
Anisotropy maps of δ^18^O and δ^2^H plotted for 2013 and 2014.

Spatial variability was quantified by means of variograms. The best fitting model was plotted together with the variogram (Fig 3). Although they exhibited a poorly pronounced moderate zonal anisotropy in the approximate direction of -40° for the δ^2^H series, overall the variogram maps may be considered isotropic (Fig 4). A steep rise of the variogram and analysis of the related scatterplots reveal a trend for the δ^2^H series and also for the δ^18^O series, although it is less noticeable in the latter case. A trend of this kind is also consistent with the above-mentioned “latitude effect”. As expected, the detrended variogram (Fig 3b and 3c) showed more variance stability. Overall, the range of autocorrelations was quite stable, ranging between 20 and 70 km for the δ^18^O and δ^2^H series, indicating high spatial autocorrelations. This result is important for future sampling design. Our field sampling was very intensive because the spatial structure of the timber isotope was still unknown, but since we reported high spatial autocorrelation up to 40 km, a lower sample density could save time while providing suitable isoscapes.

The best fitting function with the lowest AIC and RSS was the spherical model for the δ^2^H series and the Matern model with Stein’s parameterization for the δ^18^O series.

As expected, the nugget effect was higher for the δ^2^H series, with a maximum of 30 in 2014 due to the higher variance of this proxy.

**Fig 5:**
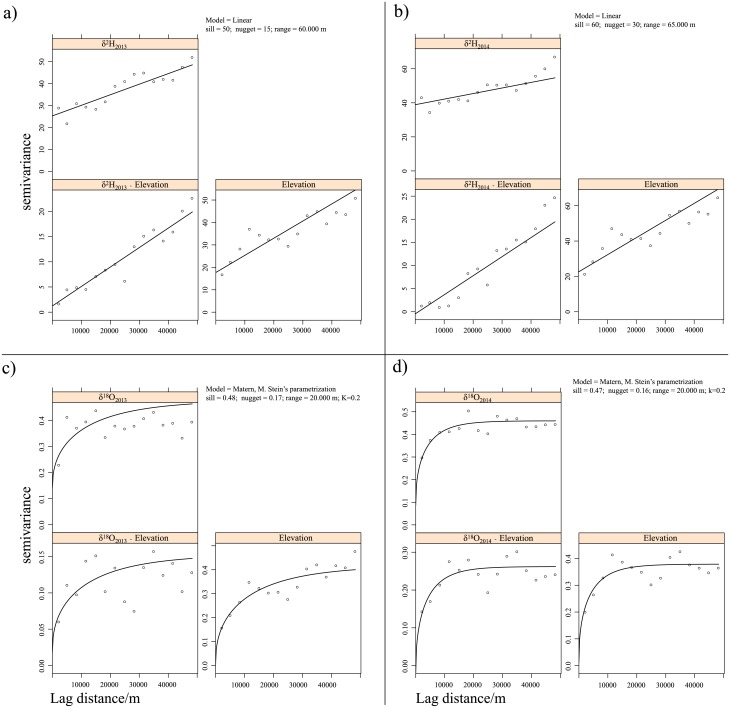
Variograms and cross-variograms of δ^2^H and δ^18^O and elevations. The lags have been binned over all directions and incremented in steps of 2,500 m. Solid lines show the models fitted to the variograms with the parameters reported in each panel.

Fig 5 shows the variograms for δ^18^O and δ^2^H with altitude as covariate and the cross-variograms with the fitted variogram model. Based on the smallest RSS, a linear model was selected for the δ^2^H series, and a Matern model with Stein’s parameterization for the δ^18^O series.

As a final step, each variogram model was used for subsequent interpolation with ordinary kriging (in the absence of a trend) or universal kriging (in the presence of a trend), while the cross-variogram was used for the cokriging model.

After cross-validation, the RMSE and MSDR of the variance revealed the performances of the different models: universal kriging (with either linear or quadratic trend) showed a slight improvement over ordinary kriging, while cokriging was the best model for all the isotope series (Table 2).

**Table 2:** RMSE (left value) and MSDR (right value) of the variance after cross-validation of the 4 kriging models.

	KriO	KriL	KriQ	CoK
**δ**^**18**^**O**_**2013**_	0.62–1.06	0.61–1.042	0.61–1.042	0.57–1.067
**δ**^**18**^**O**_**2014**_	0.64–1.02	0.64–1.04	0.64–1.04	0.55–1.04
**δ**^**2**^**H**_**2013**_	5.61–0.945	5.58–1.07	5.58–1.07	5.54–1.05
**δ**^**2**^**H**_**2014**_	6.48–1.13	6.40–1.11	6.40–1.11	6.31–0.94

KriO = Ordinary kriging

KriL = Universal kriging with linear trend

KriQ = Ordinary kriging with quadratic trend

CoK = Cokriging with altitude as covariate

Cokriging may be helpful and may prove to be a better interpolation method when a second variable related to the target variable is available. Although our sampling was dense—151 sites (5 trees per site) sampled within a range of 600 km^2^ (i.e., around 0.25 sample sets per km^2^)—the LiDAR-derived DEM had a higher spatial resolution with an average of one altitude data per m^2^. Moreover, when a covariate is available as a high-resolution grid map (as in the case of the LiDAR DEM), cokriging may also reveal local variability even when the primary variable is sparse and poorly recorded [57,66]. Together with the variogram results, this evidence is crucial in terms of saving money and time in the field sampling.

**Fig 6:**
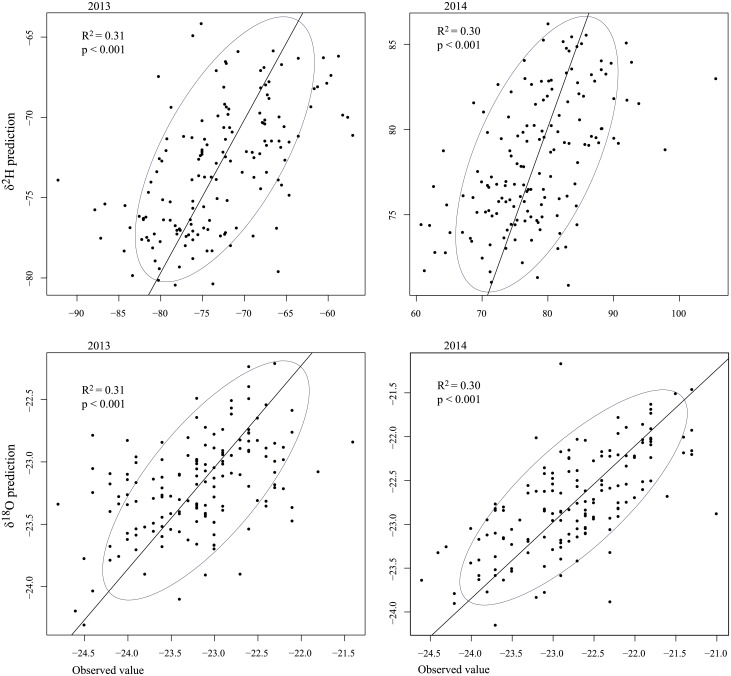
Scatter diagrams of δ^2^H and δ^18^O measured in 2013 and 2014 plotted against the values predicted by cokriging after cross-validation. The solid line represents the regression. R^2^ is reported for each plot.

To complete the picture, Fig 6 is a graph showing the observed values for δ^2^H and δ^18^O against the cross-validation predictions obtained with the cokriging model.

### δ^2^H isoscapes and δ^18^O isoscapes

**Fig 7:**
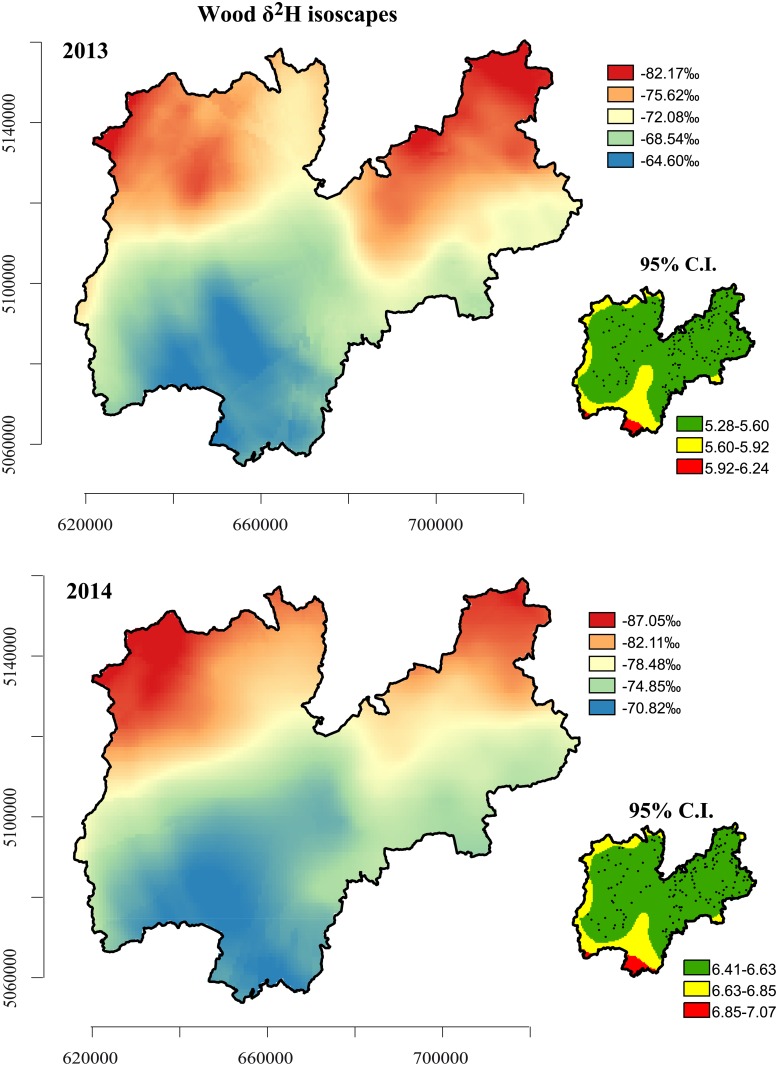
δ^2^H isoscapes and relative 95% confidence intervals for Norway spruce timber for 2013 and 2014.

Fig 7 shows the δ^2^H isoscapes with relative 95% confidence intervals for Norway spruce wood for 2013 and 2014. The 95% confidence intervals range from 5.28 to 7.7‰. As expected, the predicted error was higher at the southern and western edges than in the central and eastern areas due to the sampling frame. The magnitude of error reduction in the central and eastern regions is consistent with a wide range of sample densities in those areas. Nevertheless, this evidence is of no concern since Norway spruce trees are missing in those areas, which, for this obvious reason, were excluded from the sampling design. Higher spatial variability was found in 2014, with the 95% confidence intervals ranging from 6.41 to 7.07‰. Although the inter-annual spatial structure is basically the same, there was a greater enrichment of ^2^H in 2013 than in 2014. 2013 was drier and warmer than 2014 in this region and the positive relationship between temperature and both δ^2^H and δ^18^O satisfactorily explains this enrichment.

The isoscapes revealed the δ^2^H values to have a strong latitudinal dependency, with the northern area having lower δ^2^H values. This is consistent with the findings of Chiocchini et al. [24], who reported a slight latitudinal gradient in the δ^18^O of Italian olive oils along the Italian coast. This could also be inferred from the negative relationship between these two proxies (Table 1).

Because of the effect of altitude, the low elevations and the bottom of the Adige valley in the southern region exhibited relatively higher isotopic values. Overall, the δ^2^H spatial patterns clearly identify 3 distinct areas: a) a northern area, b) a central area, and c) a southern area, depending on the δ^2^H gradient.

There also seems to be a valley effect because the spatial structure of the isoscapes follows, more or less, the shape of the valley (Fig 1), although part of this effect can be easily explained, since the valley areas have similar rainfall patterns.

**Fig 8:**
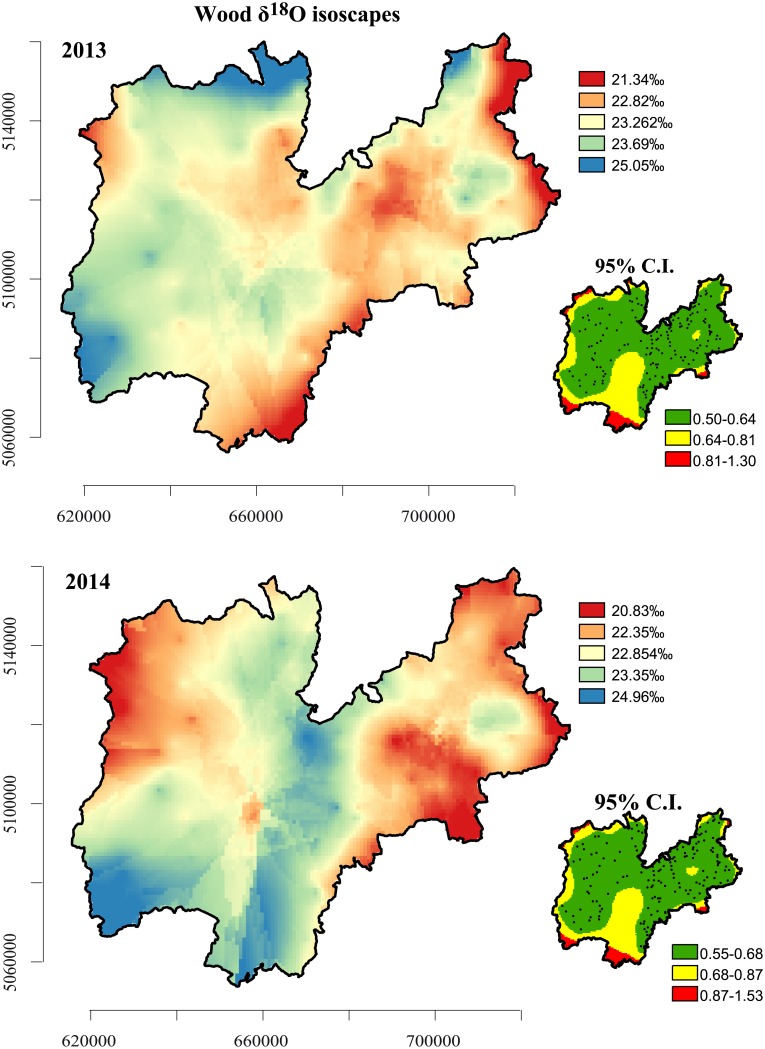
δ^18^O isoscapes and relative 95% confidence intervals for Norway spruce timber for 2013 and 2014.

Fig 8 reports δ^18^O isoscapes with relative 95% confidence intervals for Norway spruce for 2013 and 2014. The spatial structures followed similar patterns in 2013 and 2014, though to a lesser extent than the δ^2^H isoscapes and with a slightly higher predicted error reported in 2014. As with the δ^2^H isoscapes, the isoscapes were more enriched in δ^18^O in 2013 than in 2014. As with δ^2^H, the central and eastern areas of the interpolated maps had greater accuracy.

Unlike with δ^2^H, there wasn’t a very clear separation across latitudes for δ^18^O. There was a slight separation between east (higher δ^18^O) and west (more depleted) in the 2013 isoscapes, evidence, instead, for a longitude effect, although this separation appears to be less evident in the 2014 isoscapes.

High δ^18^O values characterize the valley bottom, particularly the main central valley (the Adige valley, which runs north-south through central Trentino), with a range between 24 and 25‰, depending on the year.

The spatial structures of both the δ^18^O and δ^2^H values are quite stable and are mainly influenced by altitude, although there is a certain inter-annual variability. This is not surprising, as the relationships between both δ^18^O and δ^2^H and altitude, reported in Table 1, are well known. A gradual depletion in heavy isotopes with increasing altitude is thought to be a common signal and the current explanation is that it is a consequence of isotope fractionation. Isotope fractionation, leading to preferential removal of heavy isotopes, follows the altitude gradient because the gradual condensation of atmospheric water vapour during cloud formation is altitude dependent [67].

The spatial patterns evidenced in these maps lend strong support to the isotope fractionation theory. Analyses of the two stable isotope maps superimposed reveals five main areas: the north, centre and south reflecting the δ^2^H north-south gradient (the latitude effect), and the east and west, reflecting the relationship between δ^18^O and longitude.

We point out that a multiproxy approach enhances the provenance signal, which increases the chances of clustering distinct areas and therefore of pinpointing the geographical origin of the timber. This is possible because these proxies, although similar to the mechanism of fractionation, are not subject to the same sources of ‘error’[55,68]. Because annual isotope values within a tree-ring core show high variability [55, 68], we carried out isotope measurements with annual resolution. For instance, we found an isotope spatial variability (between the northeast and southwest δ^2^H isoscapes; see Fig 7) comparable to the annual isotope variability that we found in our previous research in the same region (Trentino) [55]. For this reason, we are sure that bulk analysis of wood fails to cluster distinct areas in mountainous and small regions characterised by higher climatic and orographic variability. Accuracy is also enhanced by yearly reporting, which yields data that are more stable, less noisy and that have a lower prediction error. This is especially important for studies on a regional scale, which are affected by the low spatial variability of both stable isotopes. We should, however, mention that annual resolution is possible only after proper measurement of the tree ring and subsequent cross-dating following the standard dendrochronological protocol. At present, cross-dating is very labour intensive, but is, nevertheless, regarded as a fundamental principle of dendrochronology and is commonly applied in many forest research departments and isotope units. Cross-dating a solid sample core of wood is also a feasible means of identifying the exact calendar year in which each tree ring was formed, even where the timber was felled in the distant past and used for construction or for wooden artefacts (for example, wooden houses, log cabins, wood flooring, wooden furniture, and so on). Cross-dating fails, of course, with chipboard and, therefore, this method cannot be applied in presence of chipboard.

Even though the link between δ^2^H and δ^18^O in tree-rings and isotope ratios in the source water is well known [28,68], as a general rule, we advise caution in using isotopic maps of meteoric water (available at http://www.waterisotopes.org) as covariates for the cokriging model on a regional scale because of their coarse grid resolution. Moreover, these maps do not show the clear latitude trend of δ^2^H and δ^18^O in the Italian peninsula, as reported by Chiocchini et al [24] for the Tyrrhenian coast and also observed by us in the Trentino region.

## Conclusions

We were able to predict geographical provenance of wood on a regional scale through the construction of δ^18^O and δ^2^H isoscapes of wood with an annual resolution. To our knowledge, this is the first geospatial model developed for timber provenance.

Overall, we compared four interpolation methods for δ^18^O and δ^2^H predictions: a) ordinary kriging, b) two universal kriging methods which took spatial trends into account, and c) cokriging. Altitude was the best explanatory variable for both δ^18^O and δ^2^H so it was used as the covariate in our cokriging model. Like another hydrological study [45], we found cokriging the best interpolation method, as it had a lower RMSE and the MSDR closest to 1. Nevertheless, we are aware that cokriging requires modelling of both direct and cross-variograms, which is very time consuming for a large number of covariates [16].

Our original idea was to use solar radiation and canopy cover as covariates for stable isotope models, but these proxies failed as predictors of stable isotope values. Solar radiation exhibits high variability on a small scale (i.e., a high *nugget effect*) due to the presence of “light gaps” and high canopy cover variability [53], so we did not consider it to be a suitable covariate on a regional scale.

Although desirable, dense sampling such as ours can be very expensive, time consuming and often logistically unfeasible. Since we reported stable spatial autocorrelations up to 40 km, we argue that it is feasible and advisable to reduce the sample density, especially when operating on a larger scale. Cokriging may also preserve local variability because of covariate availability at a higher spatial resolution. Moreover, because of the strong dependence on altitude, we found that both δ^18^O and δ^2^H perform well in mountainous areas, despite their high climatic and orographic variability.

As a general conclusion, our study demonstrates that kriging interpolation is a very promising method for defining a protocol able to trace the geographical origin of timber in order to protect local economies and help prevent illegal logging. For example, once a provenance has been identified by the method, it will be possible to check whether the timber has been harvested in an area where forest certification is mandatory (i.e., where responsible and sustainable use of renewable forest products can be assumed).

These findings should form the basis for further studies on both national and continental scales, particularly in those countries where illegal logging is widespread.

## Supporting information

S1 Dataset LiDAR data-acquisition parameters. (TXT)

S2 Dataset R packages and R script used in this study. (TXT)

S1 Table Locations of sampling sites and related mean stable isotope values and the candidate covariates. Coordinates are expressed in metres according to the projected coordinates system WGS 84 UTM Northern Hemisphere Zone 32 (epsg: 32632, http://spatialreference.org/).
(XLS)

S2 Table Descriptive statistics of δ^2^H and δ^18^O and the covariates computed for the 151 sampling sites. (XLS)

S3 Table AIC values of the regressions between the stable isotope series and the candidate variables. (XLS)

S4 Table Correlation matrix of the ten proxies. (XLS)
